# Effectiveness of Granisetron in Prevention of Hypotension Following Spinal Anaesthesia in Patients Undergoing Elective Caesarean Section

**DOI:** 10.7759/cureus.12113

**Published:** 2020-12-16

**Authors:** Abhishek Chatterjee, Bhanu Gudiwada, Pratap Rudra Mahanty, Himanshu Kumar, Deb Sanjay Nag, Pradip Kumar Ganguly, Rajiv Shukla

**Affiliations:** 1 Anaesthesiology, Tata Main Hospital, Jamshedpur, IND; 2 Anaesthesiology, Varma Hospital, Bhimavaram, IND

**Keywords:** granisetron, hypotension, spinal anesthesia, caesarean section

## Abstract

Background and aim: Spinal anesthesia is the most common type of anesthesia administered for caesarean section and it is frequently associated with hypotension. When post-spinal hypotension is accompanied with bradycardia, the condition may become more complicated. Numerous pharmacological agents have therefore been tried for prevention of hypotension and 5HT3 antagonists are the latest in the armamentarium. However, studies have shown conflicting evidence regardings the effectiveness of 5HT3 inhibitors (ondansetron and granisetron) in preventing spinal hypotension. We have tried to address this controversy and also wanted to explore the adverse effects of granisetron on the foetus, if any.

Materials and methods: Two hundred patients were included in the study and divided into two groups of 100 patients each. Group S patients received 5ml of 0.9% normal saline while Group G patients received IV granisetron 1mg (diluted to 5ml) 10 minutes prior to administration of spinal anesthesia. Analysis of variance (ANOVA) test was used for comparing the data, Student t-test was applied to compare the difference between the two means and Chi-Square test was used to test significance of difference of proportions.

Results: The incidence of hypotension in Group S was 69%, whereas it was 37% in Group G (p<0.001), hence patients of Group S required a significantly higher (p=0.001) amount of mephentermine. Haemodynamic parameters were well maintained throughout the study period in patients of Group G. The neonatal outcome was assessed by Apgar score at 0 minutes, one minute, and five minutes after delivery, and it was comparable between the two study groups.

Conclusion: Intravenous granisetron 1mg if administered before administering spinal anesthesia can effectively attenuate hypotension in parturients without any adverse effects on the mother and the neonate.

## Introduction

The most commonly administered anaesthesia technique for conducting caesarean section is subarachnoid block (SAB). SAB, although considered safe, has a reported incidence of hypotension following the procedure as high as 20-40% in pregnant patients [1]. Similarly, bradycardia is also commonly associated with post-SAB and reported incidence is around 13% [2]. Spinal anaesthesia results in sympathetic block leading to a decrease in systemic vascular resistance and hypotension, which can be minimised or prevented by various methods like left uterine displacement [3], preloading [4], or co-loading [5] with crystalloids [4] or colloids [6], use of vasopressors like ephedrine [7], mephentermine [8], phenylephrine [9], etc. Hypotension caused by SAB is physiologically compensated by an increase in heart rate. However, if vagus nerve-mediated cardio-depressor reflex-like Bezold-Jarisch reflex (BJR) gets stimulated, then the cardiac autonomic balance gets shifted towards the parasympathetic nervous system leading to bradycardia, which further precipitates hypotension [10]. The combination of hypotension with bradycardia has been a matter of concern for all anaesthesiologists and therefore, using an effective agent to take care of BJR is of prime importance in management of hypotension following SAB. Both mechanoreceptors as well as chemoreceptors are responsible for hypotension and bradycardia after spinal blockade. Deformation of cardiac walls following hypovolemia after SAB stimulates the mechanoreceptors present in cardiac chambers which further leads to initiation of Bezold-Jarisch reflex. The chemoreceptors are activated by serotonin released from activated thrombocytes [11,12].

Owczuk et al. [13] and Sahoo et al. [14] conducted studies in human beings with ondansetron for attenuation of post-spinal hypotension by inhibiting BJR and they found it to be effective. Similarly, studies carried out with granisetron by Khalifa [15], Edalba et al. [16], Mohammadi et al. [17], and Megahed et al. [18] found that it was effective in prevention of hypotension following SAB. However, contrary to the above findings, Shrestha et al. [19], Lamichhane et al. [20], and Saberi et al. [21] found granisetron was not effective in prevention of hypotension following SAB.

Therefore, this study was undertaken with the aim of addressing the ongoing controversy regarding effectiveness of granisetron in preventing hypotension following SAB in obstetric patients undergoing caesarean section. The primary objective was to assess the effectiveness of 1mg intravenous granisetron in prevention of hypotension and the secondary objective was to assess the effect of granisetron in preventing bradycardia following spinal anaesthesia in parturients undergoing lower segment caesarean section (LSCS).

## Materials and methods

This was a prospective double-blind randomized controlled trial. A total of 200 healthy term parturients (between 20 - 35 years) undergoing elective caesarean section under spinal anaesthesia were included in the study. They were then divided into two groups with 100 patients in each group (Precision 0.05, Power of study - 80%, Incidence of hypotension after SAB - 20%). Parturients not willing to participate in this study, presence of any contraindication for spinal anaesthesia, history of allergy to study drug, patients on serotonin agonists or antagonists, and presence of any co-existing diseases were excluded from the study.

Parturients were randomized into one of the two groups using a computer-based random number generator. The study drugs were premixed to a volume of 5ml and presented as coded syringes to the anaesthesiologist who was not an investigator in the study. Group G patients received IV granisetron 1mg and Group S patients received IV 5ml of 0.9% normal saline. Both the groups received the allocated solution intravenously 10 minutes before spinal anaesthesia. All parameters were noted by an anaesthesiologist blinded to the group allocation. All parturients were kept fasting for eight hours and they were uniformly pre-medicated with ranitidine 150mg and metoclopramide 10mg orally on the morning of surgery with sips of water.

Baseline/pre-spinal (T0) vital parameters of parturients including heart rate (HR), systolic blood pressure (SBP), diastolic blood pressure (DBP), mean arterial blood pressure (MAP), and peripheral oxygen saturation (SpO2) were recorded in the operating room. Any fall in the systolic arterial blood pressure below 100mmHg or a fall in mean arterial blood pressure of more than 20% from baseline value was taken as hypotension and managed with intravenous mephentermine 6mg bolus. In the operating room, an IV line was secured with 18-G intravenous cannula and infusion of ringer lactate solution started. Routine standard monitors such as pulse oximetry, electrocardiography (ECG) and non-invasive blood pressure applied and monitored. Sub-arachnoid block was given in a sitting position with midline approach with a disposable 27 gauge Quincke spinal needle. After confirming the free flow of cerebrospinal fluid, 2.2ml of 0.5% hyperbaric bupivacaine was deposited in the subarachnoid space at the rate of 1ml over 10 seconds. Parturients were made supine after putting a sterile dressing over the skin at the lumbar puncture site. The time of intra-thecal drug deposition noted and the haemodynamic parameters were recorded. All the above-mentioned parameters were recorded at every two-minute interval till 20 minutes after SAB (T-1 T-2 T-3 and so on up to T-10) and then at every five minutes till the end of surgery (T-11 onwards) [17]. All the haemodynamic parameters were monitored again in the postoperative period (T-17) after one hour of surgery [22]. Further monitoring of all patients of both the study groups were continued as per institutional protocol in the recovery area. Furthermore, other parameters were also recorded like height of sensory block (by pin-prick at mid-clavicular line with blunt needle from below upwards) after five minutes of SAB, duration of surgery, amount of intra operative blood loss, total requirement of IV mephentermine, need of IV atropine to treat bradycardia, and Apgar score (Appearance, Pulse, Grimace, Activity, and Respiration) of the neonate (at birth, at one minute, and at five minutes).

Analysis of variance (ANOVA) was used for comparing the data, Student t-test was applied to compare the difference between two means and Chi-Square test was used to test significance of difference of proportions.

## Results

The patient characteristics like age, weight, height, body mass index were comparable in both the groups. The baseline haemodynamic parameters like the heart rate, systolic blood pressure, diastolic blood pressure, mean arterial blood pressure, and peripheral oxygen saturation was comparable in both the groups before giving spinal anesthesia.

The incidence of hypotension in Group S (69%) was more than Group G (37%) (Table 1) and the difference between the two groups was statistically significant (p<0.001) 

**Table 1:** Incidence of hypotension

Hypotension	Group S		Group G		p value
	Frequency	%	Frequency	%	
No	31	31.0%	63	63.0%	<0.001
Yes	69	69.0%	37	37.0%	
Total	100	100%	100	100%	

Use of mephentermine as vasopressor was also significantly higher in Group S as compared to Group G. None of the patients required atropine (Table 2).

**Table 2:** Use of mephentermine and atropine

	Group S	Group G	p value
	Mean ± SD	Mean ± SD	
Total Mephentermine (mg/patient)	12.27 ± 6.64	7.82 ± 2.80	<0.001
Total Atropine	Not required	Not required	–

**Figure 1:**
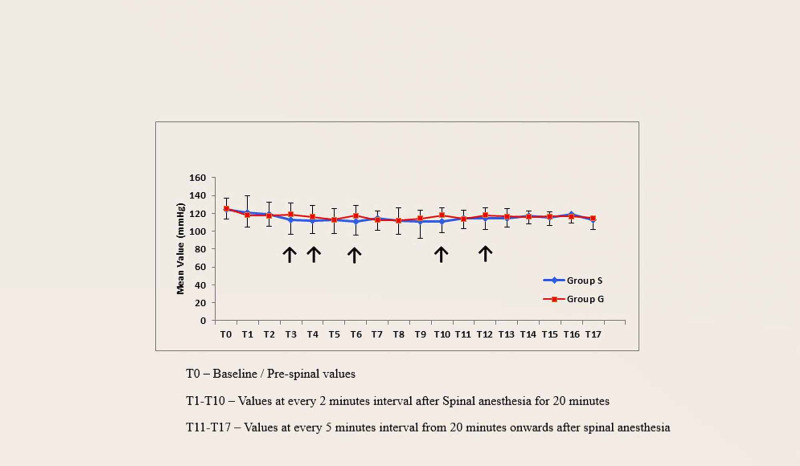
Trend of systolic blood pressure in the two groups

On comparison of the trend of systolic blood pressure between the two study groups at different time points, the difference was significant at T3 (two minutes after SAB), T4 (eight minutes after SAB), T6 (12 minutes after SAB), T10 (20 minutes after SAB) and T12 (30 minutes after SAB); p-values at these time points are < 0.05 (Figure 1).

**Figure 2:**
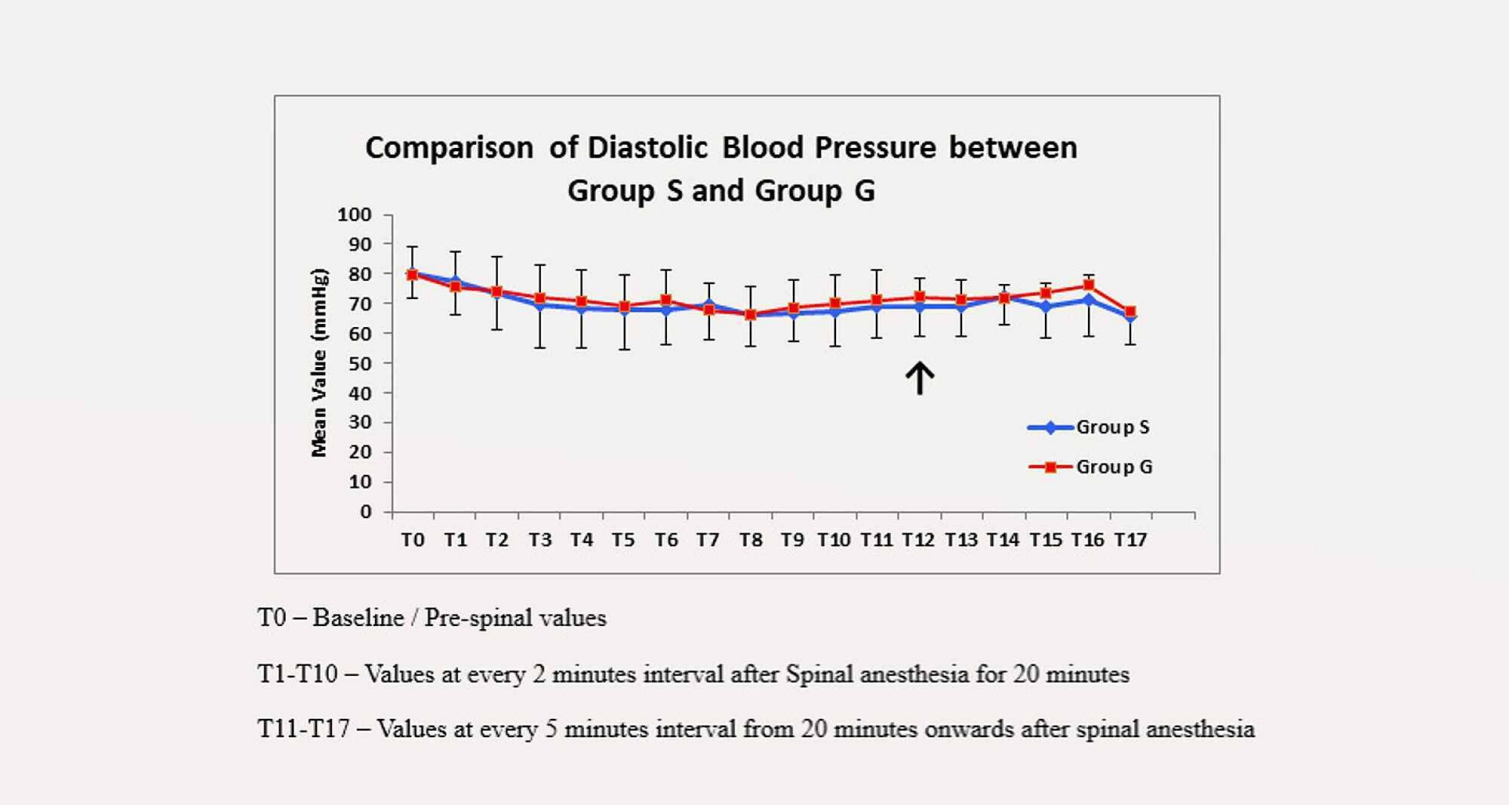
Trend of diastolic blood pressure in the two groups

The trend of diastolic blood pressure showed that except at T12 (30 minutes after SAB), the difference between the two groups was non-significant and DBP was better maintained in the granisetron group (Figure 2).

**Figure 3:**
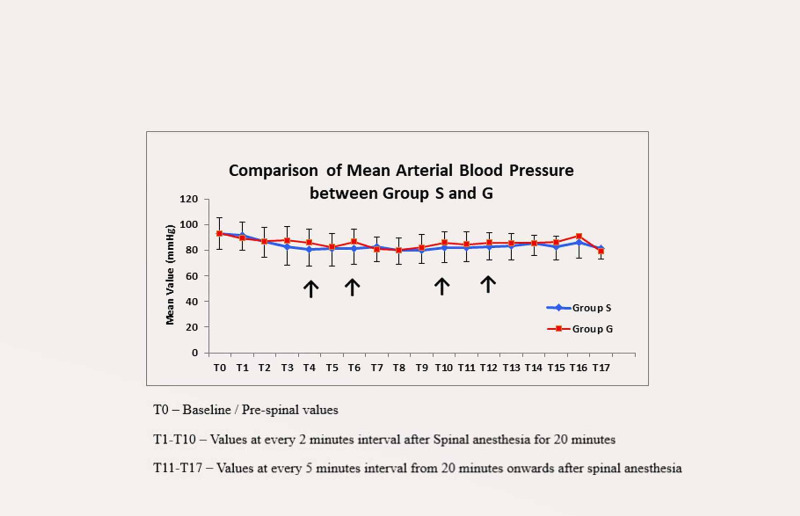
Trend of mean arterial blood pressure in two groups

The trend of mean arterial blood pressure (MAP) showed that at T4 (eight minutes after SAB), T6 (12 minutes after SAB), T10 (20 minutes after SAB) and T12 (30 minutes after SAB), the difference in MAP between two groups was significant. (p-values are < 0.05). The mean arterial blood pressure after spinal anaesthesia was also significantly better maintained in Group G at different time points in comparison to Group S (Figure 3).

There was no statistically significant difference between the study groups in heart rate after SAB (p-value > 0.05)/

There was no statistically significant difference (p = 0.101) in height of sensory block in parturients after SAB, i.e. parturients of both groups attained comparable height of sensory block. The surgical duration and amount of intraoperative blood loss between Group S and Group G were also comparable (p-values 0.740 and 0.627 respectively).

Neonatal outcome depends upon Apgar score, which was also comparable between the two study groups at 0 minutes, one minute, and five minutes after delivery.

## Discussion

Neuraxial anaesthesia remains the preferred choice for LSCS across the world and hypotension after SAB remains one of the most researched subjects in obstetric anaesthesia. Hypotension is the physiological consequence of spinal anaesthesia and can have potential deleterious maternal and foetal impact. Several methods are therefore being followed to treat hypotension after SAB like IV fluids, pharmacological agents, and physical methods like positioning. Over the last few years, different pharmacological agents are in use to treat post-spinal hypotension. Prevention of spinal hypotension in parturients where two lives are affected is a real concern for the anaesthesiologist and an effective and safe prophylactic pharmacological agent will be ideal for this purpose. With the introduction of 5-HT3 receptor antagonist in clinical practice, different authors started exploring these agents and since then the treatment options for spinal induced hypotension has been widened. Numerous articles have been published showing the efficacy of both ondansetron and granisetron for the purpose of management of post-spinal hypotension. However, a few studies have shown that granisetron is ineffective for this very purpose. We therefore tried to address this issue and have conducted a prospective, randomized, double-blind study with the aim to study the effectiveness of intravenous granisetron (1mg) in prevention of hypotension in parturients undergoing elective caesarean section under spinal anaesthesia.

In our study we observed that 67% of parturients of Group S had hypotension after spinal anaesthesia whereas only 33% of parturients developed hypotension in Group G and this difference was statistically significant with a p-value <0.001 (Table 1).

Eldaba et al. [16] in a similar study also observed a statistically significant difference with 3% incidence of hypotension in the granisetron group as compared to 64% in the normal saline group. Megahed et al. [18] and Sayed et al. [23] also observed significantly less incidence of hypotension when patients were given granisetron or ondansetron as compared to normal saline.

However, Lamichhane et al. [20] who used a similar dosage of granisetron and Shrestha et al. [19] using granisetron at 40mcg/kg did not observe a satisfactory effect of IV granisetron. Both the authors did not take intraoperative blood loss and height of sensory block into account and perhaps the patients of the granisetron group had higher sensory block or more surgical blood loss as compared to the normal saline group. In the present study, surgical blood loss, duration of surgery, and height of sensory block were similar in both groups.

In the present study mean dose of intravenous mephentermine used for the treatment of hypotension following spinal anaesthesia was significantly low in Group G compared to Group S (Table 2). Lamichhane et al. [20] and Eldaba et al. [16] had used a similar dose of granisetron in their studies. They found reduced requirement of rescue vasopressor in the granisetron group for treatment of spinal anaesthesia induced hypotension in comparison to the normal saline group. However, Mohammadi et al. [17], although they used a higher dose of granisetron (3mg) in their study, found a non-significant difference between the two study groups in relation to the need for vasopressor for management of post-spinal hypotension. Mohammadi et al. [17] did not mention factors like level of block and intravenous fluids used, which could have influenced the hypotensive episodes in the perioperative period.

Baseline systolic blood pressure (T-0) between the two study groups was comparable in the present study. After administering spinal anesthesia, the systolic blood pressure at different points of time was comparable between the two groups except for readings at the sixth minute, eighth minute, 12th minute, 20th minute, and 30th minute. Different authors studied the onset and duration of post-spinal hypotension and they observed incidence of hypotension after three minutes of SAB [24] and this hypotension persisted during the intraoperative period [25]. On comparing both the above findings, the present study showed fall in SBP was greater in the saline group until 30 minutes after SAB and granisetron had a beneficial effect against fall in SBP after SAB. Saberi et al. [21] observed that although the granisetron group showed a lesser decrease in systolic blood pressure as compared to normal saline, the difference was not statistically significant. Shrestha et al. [19] also observed a similar non-significant difference in systolic blood pressure between the two groups. Both studies did not take surgical blood loss or other influencing factors into account and therefore the findings were different when compared to our study. In our study the height of sensory block and intraoperative blood loss were comparable in both groups.

Diastolic blood pressure between the two groups was comparable throughout the study period except at the 30th minute, which indicated granisetron had a negligible effect on diastolic blood pressure as compared to systolic blood pressure. Both Lamichhane et al. [20] and Saberi et al. [21] observed similar results in diastolic blood pressure.

In the present study, baseline mean arterial blood pressure between Group G and Group S was comparable. After giving SAB, the difference in mean arterial blood pressure between the two groups was significantly different at the eighth, 12th, 20th, and 30th minute, which was similar to change in systolic blood pressure between the two groups at the same time interval. Mean arterial blood pressure takes into account both systolic blood pressure and diastolic blood pressure. As diastolic blood pressure did not show significant difference between the two groups, the changes that occurred in systolic blood pressure were reflected in a similar way and we observed similar significant changes in mean arterial blood pressure. When use of mephentermine was compared we observed significant difference between the two groups at the sixth minute, eighth minute, 12th minute, and 20th minute. Khalifa et al. [15] found that the fall of mean arterial blood pressure with granisetron and use of ephedrine was significantly low in comparison to the saline group at five minutes after SAB. Eldaba et al. [16] also concluded that granisetron significantly reduced the decrease in mean arterial blood pressure when used as a premedication before spinal anaesthesia in caesarean section and observed a prolonged median time elapse to incidence of hypotension in the granisetron group (16 minutes) as compared to the saline group (seven minutes).

In our study the baseline heart was comparable in both groups. After SAB, heart rate trend showed non-significant difference between Group G and Group S throughout the study period. Our findings of change in heart rate were similar to Lamichhane et al. [20], Mohammadi et al. [17], and Shrestha et al. [19] in two different studies; although they used higher doses of granisetron in their studies, they failed to observe significant heart rate variations.

In our study we did not find any episode of significant bradycardia in either of the groups and therefore intravenous atropine was not needed. Lamichhane et al. [20] also did not find any episode of bradycardia in either of the groups. However, Eldaba et al. [16] found significant difference in incidence of bradycardia, i.e. more number of parturients in the saline group had bradycardia in comparison to the granisetron group and they concluded that granisetron given prior to spinal anaesthesia reduced the incidence of bradycardia, perhaps due to inhibition of Bezold Jarisch reflex [16].

Incidence of hypotension after spinal anaesthesia is as high as 50-60% [1] in the obstetric population when compared to the non-obstetric population which is around 33% [2]. This difference may be influenced by many factors during preoperative period. To address this issue, we also observed total duration of surgery (skin incision to closure time), intra operative blood loss, height of sensory block, technique of subarachnoid block, etc. We administered subarachnoid block as per our institutional protocol to every parturient and we maintained same degree of Trendelenburg position in all parturients enrolled in the study.

In spinal anaesthesia when sensory block level attains T4 or above T4 dermatomal level it increases the incidence of hypotension in the obstetric population. As block level reaches at or above T4 dermatomal level, it leads to inhibition of cardiac sympathetic system which is an influencing factor on haemodynamic parameters in spinal anaesthesia [24]. So monitoring sensory block level is an important factor after administering subarachnoid block. In the current study, sensory block level attained in both groups was comparable.

Surgical blood loss also influences haemodynamic parameters and may lead to hypotension. Intraoperative blood loss along with SAB have an additive effect and may cause profound hypotension. We, therefore, explored the effect of intraoperative blood loss on incidence of post-spinal hypotension between the two groups. In our study we observed that mean intraoperative blood loss between the two study groups was also comparable.

The Apgar score describes the condition of newborns immediately after birth and when properly applied, is a tool for standardized assessment [26]. In the current study, Apgar scores at 0 minutes, one minute and five minutes were comparable between the two groups. Eldaba et al. [16] also observed similar non-significant difference in Apgar score between two groups. We therefore can say that intravenous granisetron did not have any adverse neonatal outcome.

## Conclusions

Subarachnoid block is still the most commonly performed anaesthesia technique for lower segment caesarean section. Incidence of post-spinal hypotension is high in parturients undergoing lower segment caesarean section. Intravenous granisetron 1mg if administered before SAB, besides being an effective antiemetic, can also be used to effectively attenuate hypotension in parturients without any adverse effects on mother and neonate.
